# Exploring temporal patterns of bacterial and fungal DNA accumulation on a ventilation system filter for a Singapore university library

**DOI:** 10.1371/journal.pone.0200820

**Published:** 2018-07-18

**Authors:** Irvan Luhung, Yan Wu, Siyu Xu, Naomichi Yamamoto, Victor Wei-Chung Chang, William W. Nazaroff

**Affiliations:** 1 SinBerBEST Program, Berkeley Education Alliance for Research in Singapore (BEARS), Singapore, Singapore; 2 Singapore Centre for Environmental Life Sciences Engineering (SCELSE), Nanyang Technological University, Singapore, Singapore; 3 School of Environmental Science and Engineering, Shandong University, Jinan, China; 4 Department of Environmental Health Sciences, Graduate School of Public Health, Seoul National University, Seoul, South Korea; 5 Department of Civil Engineering, Monash University, Victoria, Clayton, Australia; 6 Department of Civil and Environmental Engineering, University of California, Berkeley, CA, United States of America; Wadsworth Center, UNITED STATES

## Abstract

**Introduction:**

Ventilation system filters process recirculated indoor air along with outdoor air. This function inspires the idea of using the filter as an indoor bioaerosol sampler. While promising, there remains a need to investigate several factors that could limit the accuracy of such a sampling approach. Among the important factors are the dynamics of microbial assemblages on filter surfaces over time and the differential influence of outdoor versus recirculated indoor air.

**Methods:**

This study collected ventilation system filter samples from an air handling unit on a regular schedule over a 21-week period and analyzed the accumulation patterns of biological particles on the filter both quantitatively (using fluorometry and qPCR) and in terms of microbial diversity (using 16S rDNA and ITS sequencing).

**Results:**

The quantitative result showed that total and bacterial DNA accumulated monotonically, rising to 41 ng/cm^2^ for total DNA and to 2.8 ng/cm^2^ for bacterial DNA over the 21-week period. The accumulation rate of bacterial DNA correlated with indoor occupancy level. Fungal DNA first rose to 4.0 ng/cm^2^ before showing a dip to 1.4 ng/cm^2^ between weeks 6 and 10. The dip indicated a possible artifact of this sampling approach for quantitative analysis as DNA may not be conserved on the filter over the months-long service period. The sequencing results indicate major contributions from outdoor air for fungi and from recirculated indoor air for bacteria. Despite the quantitative changes, the community structure of the microbial assemblages was stable throughout the 21-week sampling period, highlighting the robustness of this sampling method for microbial profiling.

**Conclusion:**

This study supports the use of ventilation system filters as indoor bioaerosol samplers, but with caveats: 1) an outdoor reference is required to properly understand the contribution of outdoor bioaerosols; and 2) there is a need to better understand the persistence and durability of the targeted organisms on ventilation system filters.

## Introduction

In buildings with a centralized ventilation system, a fibrous filter in the air-handling unit (AHU) is used as a first line of defence for protecting equipment (and the indoor environment) from particulate matter. The filter typically treats a mixture of outdoor air and recirculated indoor air (up to 90% is recirculated, depending on climate and weather conditions), before controlling temperature and humidity, and then supplying the processed air to the served indoor spaces. Most of the air entering and contained within a mechanically ventilated building passes through the ventilation system. Such an arrangement inspires the idea for indoor air quality researchers to use ventilation system filters as time-integrated indoor air samplers that can be acquired with relatively little effort [1]. Studies that assess biomass collected on ventilation system filters have begun to emerge in the last decade [2–5].

Although this sampling approach appears promising, few studies have systematically investigated the factors that could limit its accuracy. Based on currently available knowledge, we identify three factors that could substantially affect the interpretation of indoor microbiology results in studies that utilize ventilation-system filters. The first factor is filter efficiency. Ventilation system filters vary widely in their single-pass efficiency when they are new [6]. Furthermore, as noted by Haaland and Siegel [1], filter efficiency can vary with time (and not necessarily monotonically) as particulate matter accumulates. As a result, biological particles could effectively be sampled at variable rates throughout the period of filter use, potentially causing errors in quantitative analysis and interpretation. A second factor concerns the conservation of DNA (or other bioaerosol analytes) on the filter. The ventilation system filter processes air at a high flow rate, only operates for part of the time, and is deployed for a relatively long period (up to 6 months or more). It is unknown whether DNA that accumulates on the filter during operation is conserved until the time of collection and analysis. The shear stress from air flow and/or vibrational motion induced by the fan could cause microbial material to become dislodged from the filter. Also, with exposure to temporally varying temperature and humidity, DNA could degrade or be amplified by microbial growth, causing errors in interpreting the analysed results. One study has shown that long sampling duration on a filter could damage a certain type of Gram-negative bacteria, compromising the subsequent analysis steps [7]. It has also been reported that biomass on AHU filters may remain viable and release metabolic byproducts [8], a finding that raises the possibility of microbial replication, potentially causing bias (overestimation) in determining the actual DNA concentration sampled from the processed air. A third important factor is the influence of both the recirculated indoor air and outdoor air on different biological entities. As most ventilation systems process a mixture of outdoor and recirculated indoor air, there is a need to consider how to properly interpret data with regard to the potential contributions of both outdoor air and indoor emissions on the microbial composition measured on ventilation system filters.

This study builds on and extends our recent effort that utilized a cross-sectional approach in investigating biomass accumulation on ventilation system filters [9]. In that study, filters from different AHUs with similar operational parameters were collected at the end of their service period (i.e., after 3 months since installation). Analysis of biomass accumulated on filters was shown to correspond reasonably well to the level of occupancy in the served indoor environments. The present study aims to contribute new knowledge about the aforementioned factors by collecting ventilation system filter samples from a single AHU multiple times on a regular basis over a 21-week utilization period. Using DNA-based analysis, we perform DNA sequencing for microbial community profiling and quantitatively trace the accumulation profile of different types of biological material (total, fungal, and bacterial DNA) on an air-handling unit filter as it functions normally. In addition, indoor occupancy data and outdoor reference samples were collected to assess the relative influence of indoor and outdoor contributions to biomass accumulation. Ultimately, the findings are expected to provide insights for future research with regards to using ventilation system filter analysis to study the microbiomes of built environments.

## Materials and methods

### AHU filter sampling information and occupancy count

Pieces of a ventilation system filter from an in-use panel filter were systematically collected from an air-handling unit in a library at Nanyang Technological University, Singapore. Among the possible locations, the library is deemed suitable for the purpose of this study for two main reasons. First, the library is always open with the same schedule during both vacation and semester period, hence the AHU operates the same way during both periods. Second, notable fluctuations of occupancy level are often seen in the library as it is mainly occupied by students, who are usually not present during the vacation periods. Other indoor spaces such as the classrooms in the university only had their AHU turned on when the rooms were used. Also, high fluctuation in occupancy level over time is not seen in offices as university staff work with a largely consistent schedule during both instructional sessions and vacation periods.

For large commercial buildings in Singapore, it is typical to have an AHU room dedicated as a mixing chamber for blending the recirculated indoor air with outdoor air. For consistency, the filter materials collected will hereafter be termed AHU filter samples. This AHU processes a mixture of recirculated indoor (90%) and outdoor (10%) air with an approximate total air flow rate of 7.8 m^3^/s. The AHU operates 71 hours/week with longer operating hours on weekdays than on weekends. The AHU serves a reading hall in the library with an approximate volume of 4170 m^3^ (area = 1250 m^2^, height = 3.2 m). The reading hall is carpeted and furnished as a typical library with tables and chairs plus book shelves.

The AHU filter was rated MERV-8 (pleated polyester) and had a total operational area (with pleats flattened) of 6.4 m^2^. Filter samples were collected with permission from the library and the university’s management office. In total, 11 sampling sessions were carried out from November 2014 to May 2015 (21 weeks). For each sampling session, at least five filter pieces of the same size (10 cm^2^) were collected for primary sampling as well as back up and replication purposes. For weeks 1 through 4 of the campaign, sampling was performed every week. For the subsequent 10 weeks, samples were collected every second week (i.e., weeks 6, 8, 10, 12 and 14). Two final sets of samples were collected at the end of weeks 18 and 21, respectively. All of these sampling times are referenced to the installation of a new bank of filters in the AHU. After each collection, the gap created was immediately covered with a new filter panel, allowing the AHU to continue its normal operation. (See S1 File for more information about the sampling effort.) Each sampled filter piece was transferred into a sterile 50 mL falcon tube and transported to the lab on ice for extraction. Pieces of a new, unused AHU filter were also cut and extracted for blank sample analysis.

**Fig 1:**
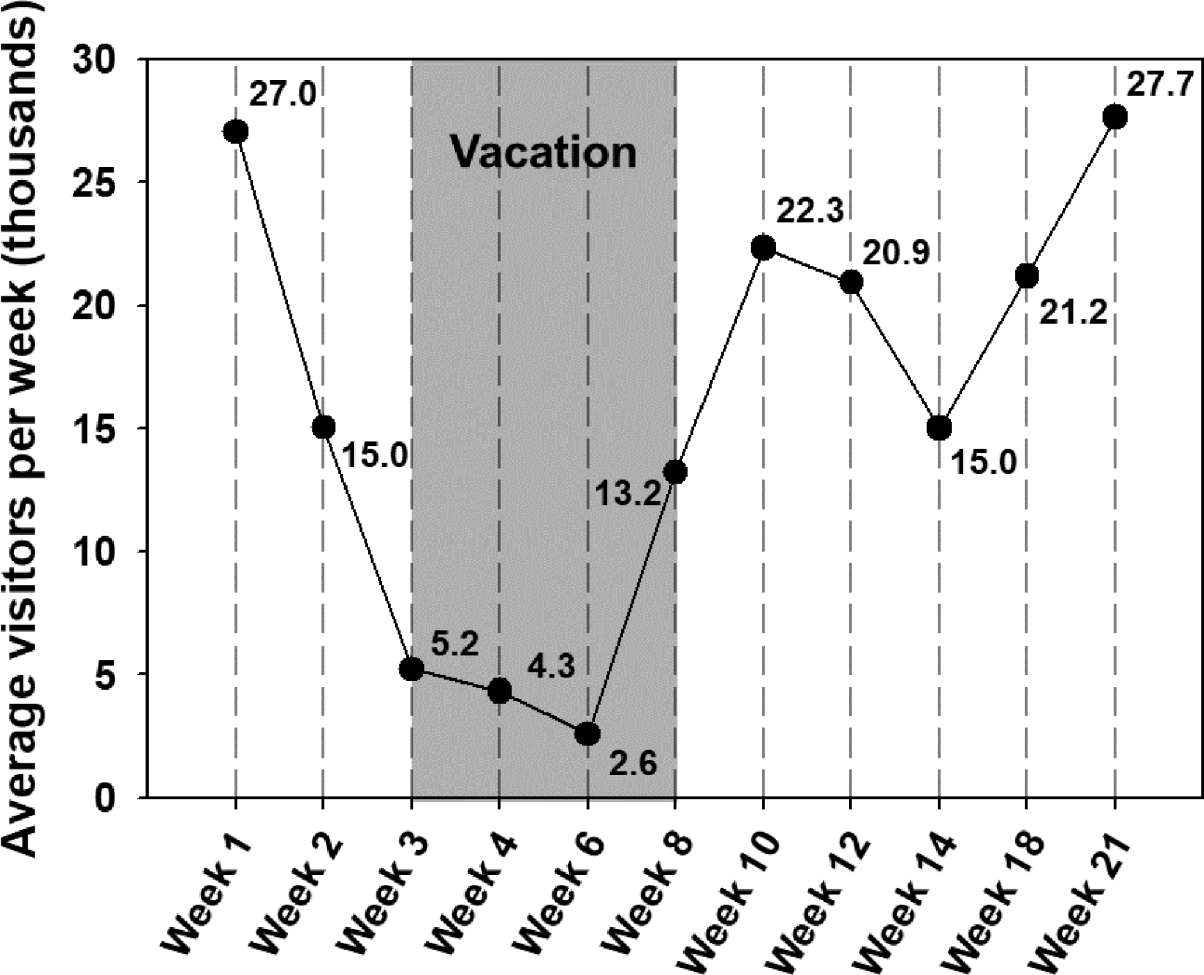
Weekly occupancy count. Average number of weekly visitors in the library. Weeks 3–8 were a vacation period; however, the library remained open for use. During the other weeks, classes were in session.

Information about occupancy was obtained through an automatic counter at the gate of the library. This sensor counts the number of persons passing through the gate towards the library every day. Fig 1 displays the weekly number of visitors over the 21-week sampling campaign. From week 4 to week 21, because filter samples were collected at intervals of more than one week, the number of average weekly visitors (visitors/week) in this period were calculated by taking the weekly average for each duration between two sample collections.

### DNA extraction and quantification

Each of the 10-cm^2^ filter samples was placed into a 5-mL bead beating tube of the MO BIO Power Water kit (MO BIO, Carlsbad, CA, USA) for DNA extraction. The DNA extraction steps followed the manufacturer’s protocol with some modifications to improve DNA yield [7]. Briefly, the modifications included vortexing for two minutes after adding the preheated solution PW1 (the lysing agent), followed by water-bath sonication at 65°C for 30 minutes. After sonication, the samples were vortexed again for 5 minutes as recommended by MO BIO. The rest of the extraction steps followed the MO BIO original protocol. Extraction replicates (three replicates for each sample) were separate 10-cm^2^ filter pieces that were cut from the same filter panel. As a negative control, clean (unused) AHU filters were briefly installed in the AHU at the beginning of the sampling campaign, then immediately removed and extracted using the same protocol.

The total DNA in each sample was measured by the Qubit high sensitivity (HS) dsDNA kit (Qubit 2.0, Invitrogen, Life Technologies, Carlsbad, CA, USA), a fluorescent dye dsDNA binding assay.

Bacterial and fungal DNA levels were quantified by means of qPCR (Step One Plus, Applied Biosystems, Life Technologies, Carlsbad, CA, USA) using Applied Biosystems Taqman fast advanced master mix and a set of universal bacterial and fungal primers and probes. The run conditions for qPCR analysis consisted of a holding stage (95°C for 20 seconds) and a cycling stage (1 second of 95°C and 20 seconds of 60°C repeated for 35 cycles). The primers for bacteria targeted the 338 to 805 region of the 16S rDNA with the forward primer BAC338F (5’-ACTCCTACGGGAGGCAG-3’), the reverse primer BAC805R (5’-GACTACCAGGGTATCTAATC-3’), and the Taqman probe BAC516F (6FAM–TGCCAGCAGCCGCGGTAATAC-3’-BBQ). The primers used for fungi targeted the fungal 18S rRNA gene [10] with the forward primer FungiQuant-F (5’-GGRAAACTCACCAGGTCCAG-3’), the reverse primer FungiQuant-R (5’-GSWCTATCCCCAKCACGA-3’) and the Taqman probe FungiQuant-PrbLNA (6FAM-TGGTGCATGGCCGTT-3’-BBQ).

We established standard curves for bacteria and fungi with DNA extracts from *Escherichia coli* (ATCC 15597) and *Aspergillus fumigatus* (ATCC 26644), respectively. DNA was extracted from suspensions of both organisms in water with an initial concentration of 40 ng/μL for *E*. *coli* and 5 ng/μL for *A*. *fumigatus*. A dilution series (10^−1^, 10^−2^, 10^−3^, 10^−4^, 10^−5^) was then prepared. The DNA concentrations of the suspensions were measured by both fluorometry (ng DNA/μL) and qPCR (*Ct*) to establish standard curves with which to relate measured *Ct* values to DNA concentrations. Final DNA concentrations are presented in terms of DNA mass per AHU filter surface area (ng DNA/cm^2^).

### Weekly DNA gain/loss rate vs. weekly occupancy

The presence of human occupants in an indoor environment has been associated with the abundance and diversity of indoor airborne microbiota [11–13]. To gauge the effect of occupancy on biomass accumulation on the AHU filter, the weekly DNA change rate was plotted against the weekly number of visitors. Weekly DNA change was assessed as the difference between the DNA concentration per filter area (ng cm^-2^) measured at a given sampling time, minus the same measure from the most recent previous sampling time, normalized by the number of weeks between sampling times. Consequently, the units of measure for this parameter are ng cm^-2^ wk^-1^. The Pearson correlation coefficient (*R*) was calculated for each correlation of DNA change rate versus weekly number of visitors and each correlation was tested for statistical significance at *p* < 0.05.

### Outdoor air sampling

Outdoor reference samples were collected at a nearby location to assess the influence of outdoor air on the microbial DNA accumulating on the AHU filter. The outdoor air samples were collected on three occasions: week 6, week 18 and week 20. The outdoor air was sampled at an open balcony outside the library by drawing the air through filter media using a vacuum pump with a flow rate of 1000 L/min (face velocity at 1.8 m/s). A new, unused AHU filter with the same MERV-8 grade as in the library was used for this sampling effort. Each of the three sampling sessions had a duration of 24 hours. DNA was extracted from each of the three filter samples and pooled [7] to concentrate biomass and improve detection, resulting in one average outdoor reference that is intended to be representative of the 21-week study period. The outdoor reference sample was submitted for DNA sequencing.

### DNA sequencing

In all, twelve filter samples were sequenced: eleven AHU filters plus one outdoor reference sample. Bacterial 16S rDNA and fungal ITS regions were targeted for DNA sequence analysis. The sequences of universal bacterial primers were 5’-CCTACGGGNBGCASCAG-3’ for the forward primer 341f and 5’-GACTACNVGGGTATCTAATCC-3’ for the reverse primer 805r [14]. The sequences of universal fungal primers were 5’-CTTGGTCATTTAGAGGAAGTAA-3’ for the forward primer ITS1 and 5’-GCTGCGTTCTTCATCGATGC-3’ for the reverse primer ITS2 [15]. These primer sequences were attached with the adapter sequences for Illumina MiSeq. Index PCR was subsequently performed with the Nextera XT index kit (Illumina, Inc., San Diego, CA, USA). Amplicon purification was performed after each PCR with the AMPure XP beads (Beckman Coulter, Indianapolis IN, USA) to remove primers and dimers. The concentration of each library was measured and normalized to 4 nM with 10 mM Tris-HCL (pH = 8.5). The libraries were pooled with an internal control PhiX (30%) before loading onto the sequencer. The pooled library was sequenced (2 × 300 bp) by means of the Illumina MiSeq with the v3 reagent (Illumina, Inc.).

The poly N tails were removed from the sequences by Trimmomatic version 0.33 [16]. The read 1 and 2 sequences were joined with a minimum allowed overlap of 10 bp in QIIME [17]. Sequences shorter than 100 bp were removed by the Galaxy Tool version 1.1 [18,19]. Additionally, chimeric sequences were removed in mothur v.1.25.0 [20] against the fungalITSdatabase database containing named fungal ITS sequences [21] for fungi, and against the reference Ribosomal Database Project (RDP) database [22] for bacteria.

For the fungal ITS sequences, taxonomic assignments were performed using BLASTN version 2.2.28+ [23] against fungalITSdatabaseID containing named fungal ITS sequences [21] and classified using FHiTINGS [24]. Prior to diversity analyses, 16876 sequences were subsampled from each library and binned into operational taxonomic units (OTUs) at 97% sequence identity using mothur version 1.25.0 [20]. The Chao 1 estimators (species richness) and Shannon indices (species richness and evenness) were subsequently calculated.

For the bacterial 16S rDNA sequences, taxonomic assignments were performed using the RDP naïve Bayesian classifier [22] with 0.8 as a confidence cutoff value. The Chao1 estimators and Shannon indices were also calculated based on 3565 sequences clustered into 97% OTUs subsampled from each library.

For β-diversity analysis between selected groups of samples, the Jaccard indices (Jclass, community membership) and the Yue and Clayton theta similarity coefficients (ThetaYC, community structure) were calculated. The parsimony tests (P-tests) were performed using mothur v1.25.0 based on these distance indices to gauge the similarity in microbial assemblages between groups of samples. Any *p*-value smaller than 0.05 was considered statistically significant.

All unprocessed raw sequences have been uploaded to NCBI under the bio-sample accession SAMN07514062-73 for bacteria sequences and SAMN07514074-85 for fungi sequences.

## Results and discussion

### Blank samples analysis

The DNA concentrations from the blank samples were all below the detection limit of the Qubit and no amplification was detected in the qPCR run with 35 thermal cycles, suggesting that there was no significant contribution of biomass from the maintenance company personnel during filter replacement, from the researchers during sampling and analysis of the filter pieces, or from the AHU filter materials themselves. In addition, all DNA samples were also tested for possible PCR inhibitions by observing the change in *Ct* values upon dilution of the DNA concentrations. No significant inhibition was found in the samples (S2 File).

### Time-series profile of DNA accumulation on AHU filter

**Fig 2:**
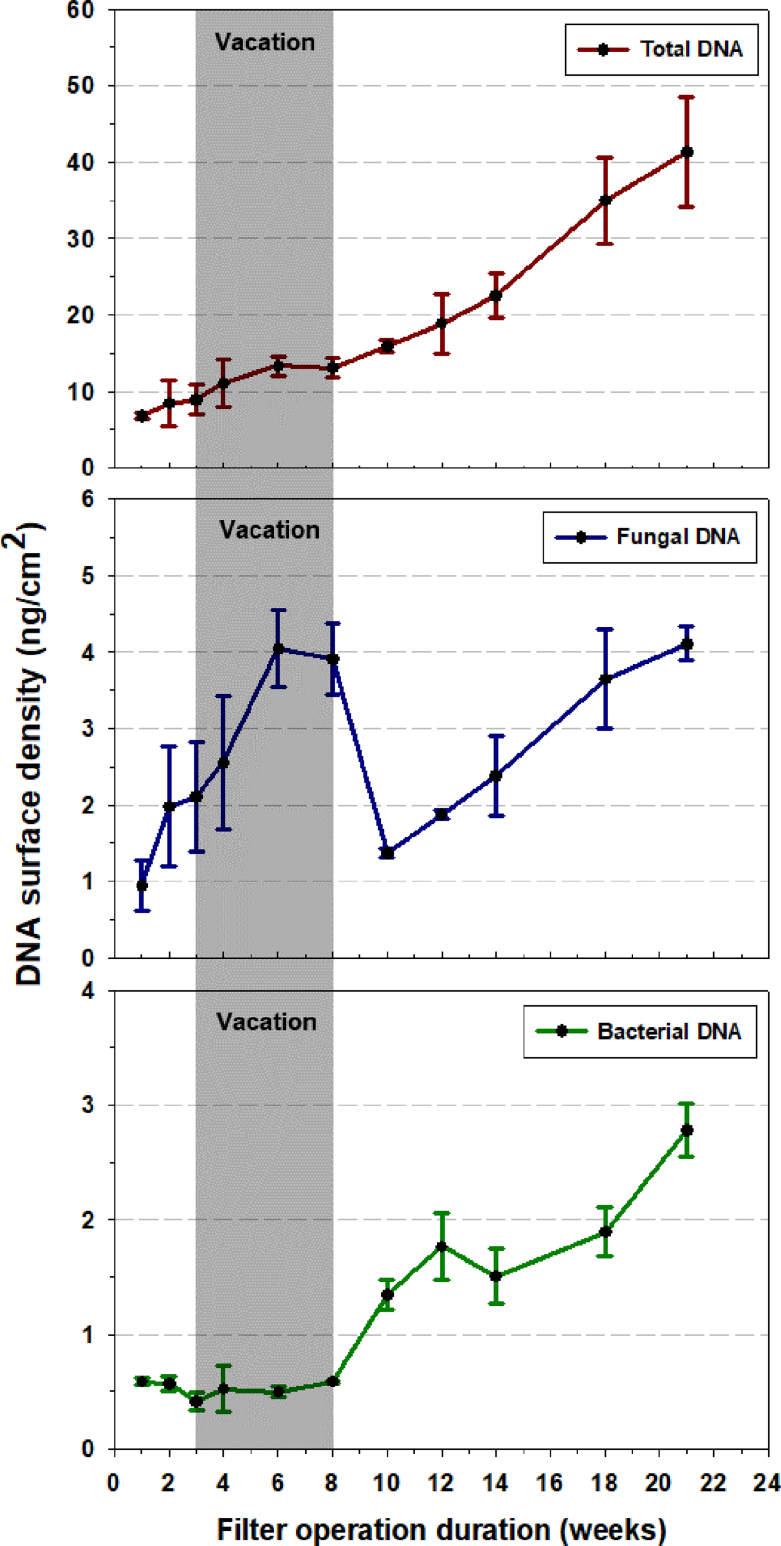
Time-series profile of DNA accumulation on AHU filter. Total (red), fungal (blue) and bacterial (green) DNA surface densities sampled over the course of the 21-week study period. The error bars represent standard deviations based on three biological replicates.

The absolute quantification data (fluorometry and qPCR) displayed in Fig 2 indicate that each broad category of biomass exhibits a distinct pattern of accumulation on the filter. Total DNA increases monotonically throughout the sampling campaign, from 6.8 ng/cm^2^ at the end of week 1 to 41 ng/cm^2^ at week 21. Fungal DNA rose quickly from 1.0 ng/cm^2^ to 4.0 ng/cm^2^ early in the campaign. Then, surprisingly, the fungal DNA surface density showed a sharp dip to 1.4 ng/cm^2^ between weeks 6 and 10, before resuming a steady rise, reaching 4.1 ng/cm^2^ at week 21. Bacterial DNA loads were relatively constant at 0.5–0.6 ng/cm^2^ for the first 8 weeks, but then generally increased with time for the remainder of the campaign, reaching a peak of 2.8 ng/cm^2^ at the end.

**Fig 3:**
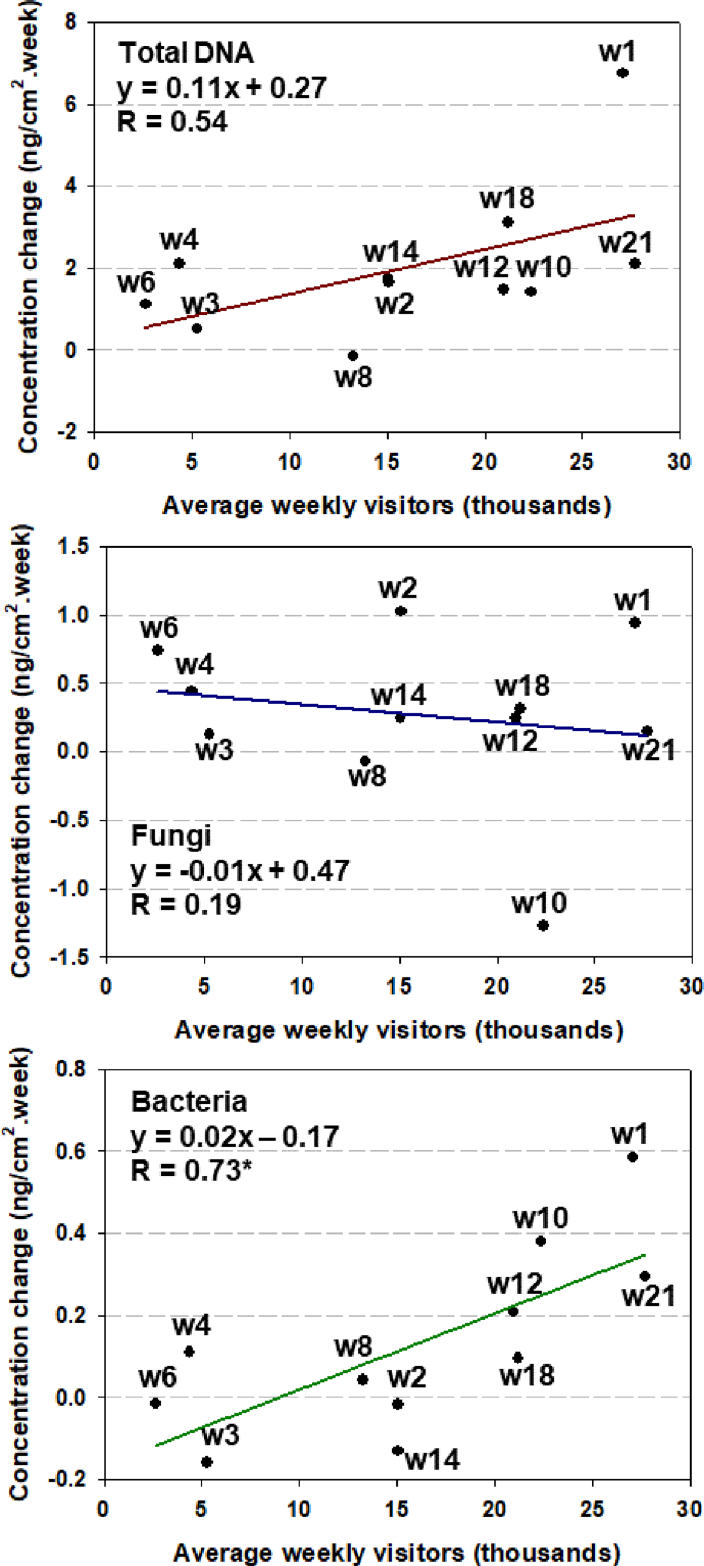
Correlation between DNA change rate and weekly number of occupancy. Linear regressions for total, fungal and bacterial DNA; * denotes statistical significance for the linear regression (*p* < 0.05).

Among the three biological entities, the rate of bacterial DNA accumulation on the AHU filter displayed the strongest correlation with occupancy. As shown in Fig 3, the average weekly changes in bacterial DNA were significantly (*p* < 0.05) correlated to weekly number of visitors in the library. Total DNA showed a weaker correlation with occupancy that was not statistically significant (*p* > 0.05). Fungal DNA accumulation on the AHU filter did not correlate with occupancy.

The strong correlation between the amounts of bacteria found on the filter and occupancy level supports an interpretation that primary indoor emission sources, mainly those associated with occupants and their activities, dominate the contribution of bacteria to the AHU filter, transmitted through the recirculated indoor air. In contrast, the contribution of fungi and other non-microbial DNA (animal or plant DNA) from the indoor environment could be overshadowed by biomass that accumulates on the filter from other sources, such as the outdoor air intake. This finding suggests that, for similar ventilation system configurations, a protocol based on AHU filter sampling could be a good probe for characterizing indoor bacteria. However, in the case that other biological entities such as fungal or total DNA are targeted in such a study, an outdoor reference is recommended to assess the relative contribution of indoor and outdoor air to the accumulation on the AHU filter.

Another important point to highlight is the possibility for a portion of DNA that is trapped on the filter to either degrade or dislodge from the filter during its continued deployment. A major advantage of DNA-based analysis for studies of the indoor microbiome is its independence of the viability or culturability of the captured organisms. DNA can be extracted and analysed even when the target organism is not alive. Nevertheless, a concern remains: would the DNA measured at the end of the sampling period be the same as that accumulated from the air passing through the filter?

One indication highlighting the validity of this concern is the substantial reduction in fungal DNA, especially evident at week 10 (Fig 2). If collected fungal DNA were strictly conserved, then its abundance on the AHU filters should increase monotonically throughout the period of deployment. We have carefully reviewed our sampling and analysis methods and do not see a flaw in them that could account for the dip exhibited in the fungal DNA time series presented in Fig 2. (Please refer to the S2 File for a report of our investigation into the possibility of inhibition in the qPCR analysis, particularly for the weekly samples involved in the dip.) Indeed, the pattern of a substantial decline over two successive samples (weeks 8 and 10) followed by steady subsequent increases through the rest of the campaign suggests that there were real losses of measurable fungal DNA from the AHU filter during weeks 6–10.

In addition to the apparent loss of measurable fungal DNA, it also appears that the fungal deposition rate on the filter may have been systematically lower during weeks 6–10 than for the rest of the sampling campaign. Three lines of evidence support this inference. The first is based on our findings for both microbial community and absolute quantification analysis. As detailed in the next subsection, only a small part (7% of average combined relative abundance) of fungal DNA was identified as being of primary indoor origin and the accumulation rate of fungal DNA showed little response to changes in occupancy (Fig 3). This evidence suggests that fungal DNA on the AHU filter may have originated mainly from the outdoor air intake.

The second line of evidence derives from a follow-up inquiry with the university facilities office. After the study period, we inquired of the building management office about the consistency of AHU operation practices during the campaign. We were told that they conducted experiments during a month-long period (January 2015, week 6 to week 10) to assess whether building energy usage could be reduced. Because the occupancy of that period was lower (owing to vacation and the beginning of the semester), it was assumed that less fresh air would be needed to ventilate the indoor environment. For that reason, the outdoor air supply was reduced, lowering the energy demand for dehumidifying supply air. This reduction in outdoor air intake, which was identified as the main source of fungal DNA, could have reduced the accumulation rate of fungal DNA on the filters for this one-month period. The system was returned to normal operation after week 10 (i.e., at the start of February 2015).

The third line of evidence is based on findings from an additional set of experiments, which was conducted to further investigate the possibility of DNA loss from AHU filters after collection and before analysis. In these experiments, we exposed used AHU filters to controlled conditions, such as elevated temperature, and measured the DNA before and after the exposure. The results support the inference that DNA is not always conserved on AHU filter: a portion of DNA was consistently lost after treatment. (Refer to the S3 File for a detailed report of these supplemental experiments.)

In summary, the best explanation that we can offer for the dip in the fungal DNA abundance on the AHU filter samples has two elements: (a) a lower rate of gain of fungal DNA from its main source because of reduced rate of outdoor air intake; and (b) the possibility of DNA degradation and/or loss from the filter during use. That the dip observed for fungal DNA was not also observed for bacterial DNA could be explained by the finding in the supplemental experiment (S3 File) in which the loss of fungal DNA was consistently found to be larger than bacterial DNA, suggesting that fungal DNA is more prone to the specific sampling conditions than bacterial DNA. Furthermore, the contribution from recirculated indoor air, which is identified as the main source of bacteria found on the AHU filter in the next subsection, was not reduced. No change was made in the recirculated airflow rate during the energy reducing test period. Continued accumulation of these DNA measures from indoor air may have exceeded any losses, so that net gains were consistently observed.

Future research on indoor bioaerosols should include an effort to understand more completely whether DNA that accumulates on filter media during air sampling in general and on AHU filters in particular is conserved. If DNA is not conserved on filters during sampling, it will be important for future bioaerosol studies to assess the nature and significance of DNA loss mechanisms for targeted organisms before proceeding with more extensive experimental investigations of the building factors that influence indoor air microbiota.

### Microbial diversity analysis: Predicting the source of microbial assemblages on the AHU filter

Overall, 759 fungal and 465 bacterial genera were detected from the twelve sequenced samples. The main purpose of the diversity analysis is to estimate the influence of both the outdoor air and changes in indoor condition (mainly occupancy level) on the microbial assemblages found on the AHU filter. The outdoor influence is appraised by observing the degree of similarity between the AHU filter samples and the outdoor air reference, while the effect of occupancy can be discerned by observing potential differences in microbial assemblages on the filter between the vacation period and times when classes were in session.

**Fig 4:**
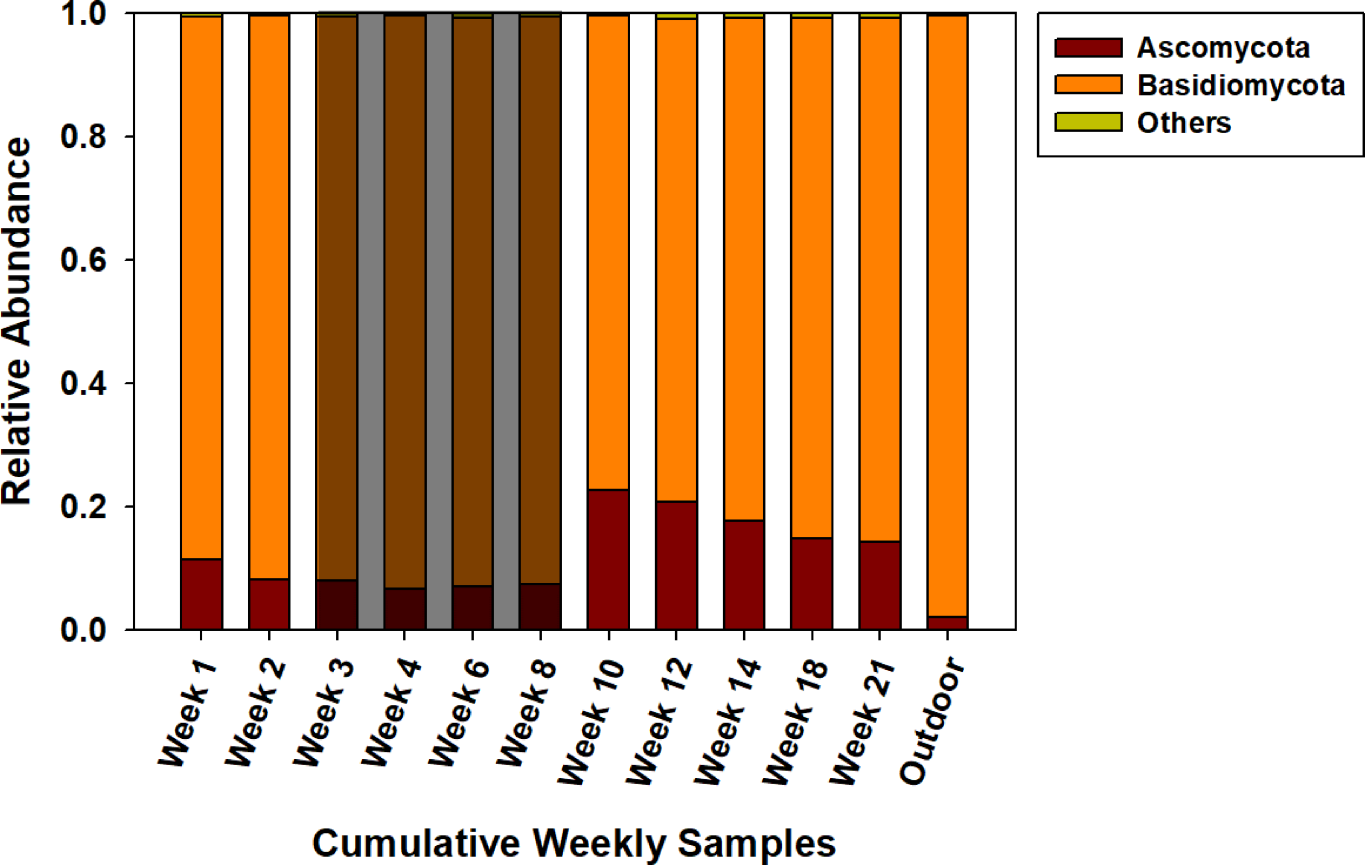
Phylum composition of fungal DNA. Composition of fungal DNA extracted from eleven AHU filter samples and one outdoor reference. The shaded area indicates the vacation period.

Fig 4 displays the finding that Basidiomycota was the dominant fungal phylum in all 12 samples with relative abundances ranging from 77% (week 10) to 92% (week 6) for the AHU filter samples and 98% for outdoor air. The phylum Ascomycota was found to be substantially present in the AHU filter samples, but not in outdoor air. The relative abundance of Ascomycota increased considerably from a combined average of 7% during vacation weeks to 16% (*p*<0.05, ANOVA) during the weeks with higher occupancy level.

**Fig 5:**
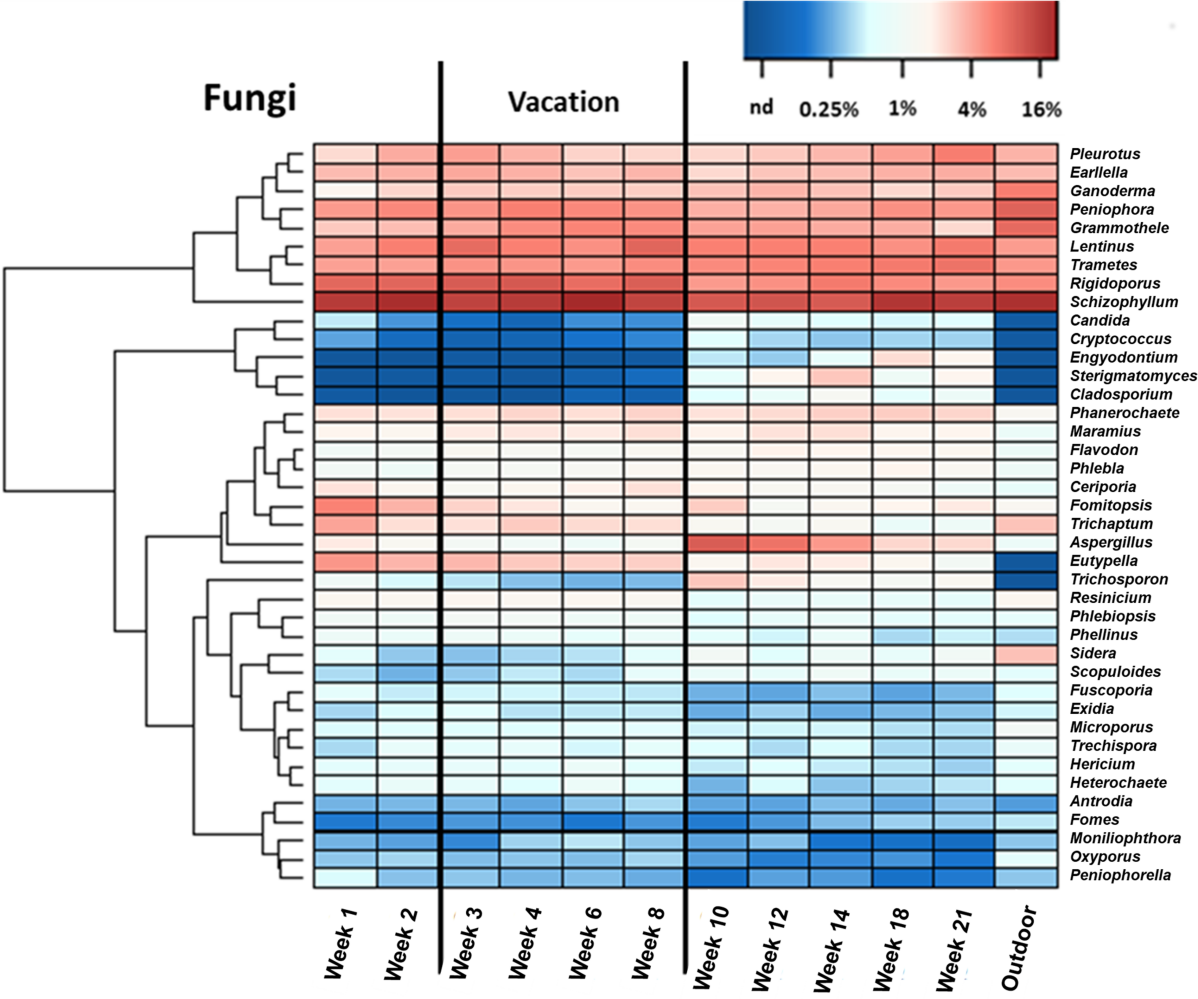
Fungal genus heatmap. Heatmap composition of the 40 most abundant fungal genera from eleven AHU filter samples and one outdoor reference sample.

A fungal genus heatmap (Fig 5) provides further insight in understanding the relative contributions of the two primary sources of biomass—outdoor air and indoor emissions associated with occupancy—found on AHU filters. The five most abundant genera found on the AHU filters and their mean relative abundances were *Schizophyllum* (16%), *Rigidoporus* (7.9%), *Lentinus* (6.5%), *Trametes* (5.7%) and *Peniophora* (5.1%). These genera are comparably abundant in the outdoor air reference. The top seven genera found on the outdoor air sample were *Schizophyllum* (20%), *Peniophora* (9.6%), *Grammothele* (8.6%), *Ganoderma* (6.6%), *Rigidoporus* (5.6%), *Trametes* (4.9%), and *Lentinus* (4.8%). The abundances of the dominant genera on AHU filters remained relatively consistent throughout the 21-week period of operation despite notable changes in occupancy level.

There was no significant difference found in terms of the community structure between the vacation and semester period (ThetaYC, *p*>0.05). (The S1 File contains β diversity plots and principal coordinate analysis plots based on the Jclass and ThetaYC metric for bacterial and fungal assemblages.) The observed similarity to outdoor air suggests a dominant contribution of outdoor fungi to the AHU filter. It must be noted, however, that while outdoor fungi could deposit on the filter directly from the outdoor air intake of the AHU, past studies have also established that fungi from outdoor environments can enter indoor spaces through leaks in the building envelope and/or via human occupants as secondary carriers (e.g., on clothing or shoes) [25,26]. Indoor fungi of outdoor origin can then become airborne indoors via shedding and resuspension and subsequently deposit on the filter through recirculated indoor air. The distinction between these two pathways for fungal DNA to accumulate on AHU filters is important with regard to exposure of building occupants: capture from outdoor air is a protective influence of filtration whereas capture from indoor releases is indicative of a potential for occupants to experience inhalation exposure.

Although there was no statistically significant difference in community structure (ThetaYC *p*>0.05), a statistically significant change in fungal community membership (Jclass metric *p*<0.05) was found in the AHU filter samples between the vacation and active semester periods. As displayed in Fig 5, this change is thought to be driven by the shifting relative abundances of previously reported human related fungal genera, such as *Candida*, *Cryptococcus* and *Trichosporon* [12], common fungi, such as *Aspergillus* and *Cladosporium* [27–29] and other genera, such as *Engyodontium* and *Sterigmatomyces*, whose relative abundance appeared to vary according to indoor occupancy level. In the weeks with higher occupancy (weeks 1–2 and 10–21), as compared with the lower-occupancy period (weeks 3–8), mean abundances of the genera increased significantly from 0.2% to 0.6% for *Candida*, 0.1% to 0.3% for *Cryptococcus*, 0.3% to 1.5% for *Trichosporon*, 1.1% to 4.5% for *Aspergillus* and 0.1% to 0.6% for *Cladosporium* (all *p*<0.05 ANOVA). Furthermore, six of these seven genera were not detected in outdoor air. Among the seven genera, only *Aspergillus* was detected in outdoor air, and with lower (0.9%) relative abundance than in the AHU samples. This evidence suggests that these fungal genera mainly reached the filter from primary indoor sources, predominantly associated with occupants. Compared to the group of dominant genera from outdoor origin, however, these genera only accounted for a small percentage of total fungi found on AHU filter samples, with an average combined abundance of 7.1%.

**Fig 6:**
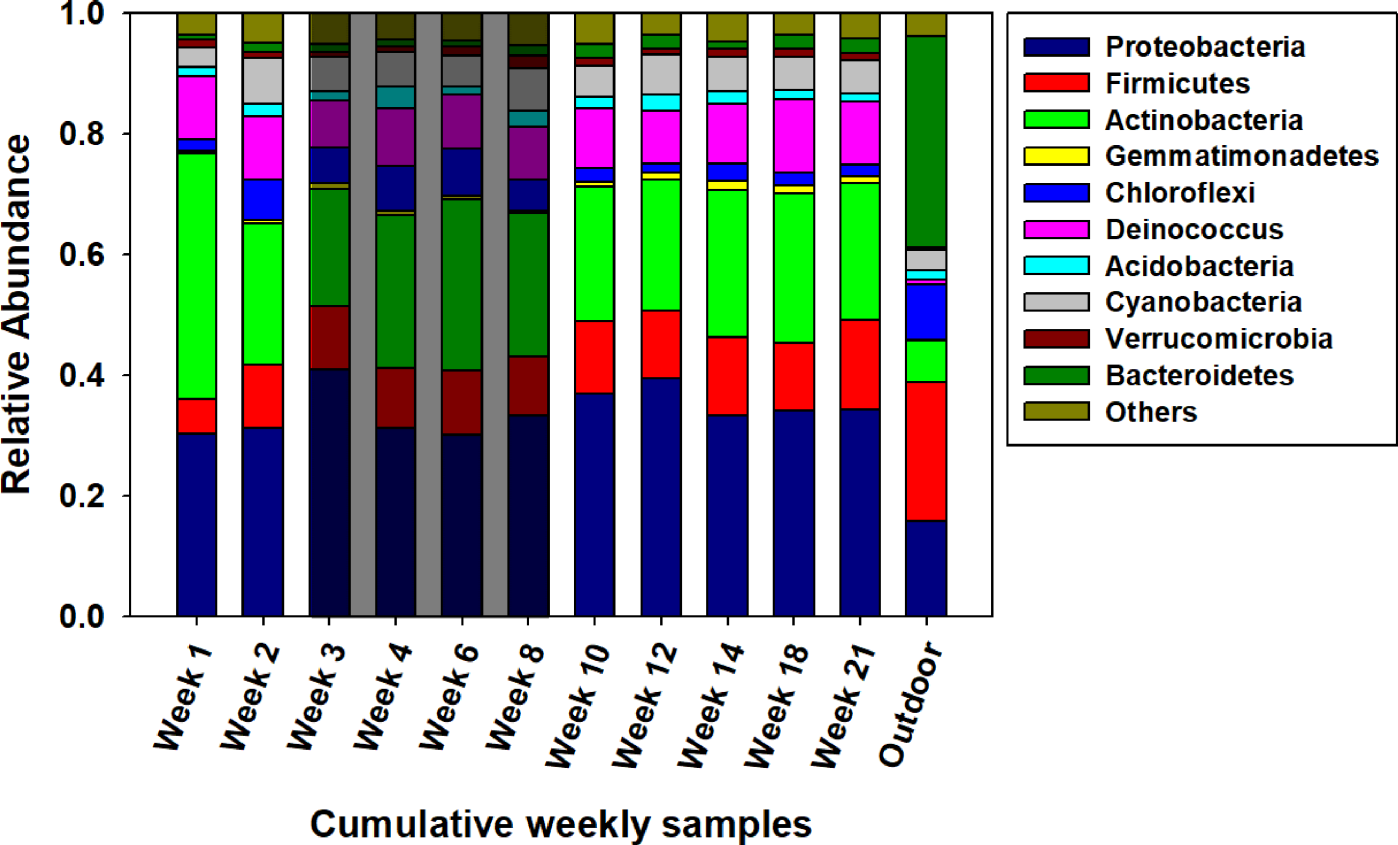
Bacterial phylum composition. Composition of bacteria extracted from 11 AHU filter samples and 1 outdoor reference. Weeks 3–8 correspond to the vacation period.

In contrast to fungal DNA, notable differences for bacterial phylum composition were seen between the outdoor air reference and the AHU filter samples. As shown in Fig 6, the three most abundant bacterial phyla on the AHU filter samples and their mean relative abundances were Proteobacteria (33%), Actinobacteria (26%) and Firmicutes (10%). Proteobacteria and Actinobacteria were considerably more abundant in the AHU filter samples than on the outdoor air samples where they comprised 16% (Proteobacteria) and 7% (Actinobacteria), respectively. Conversely, Firmicutes (23%) was relatively more abundant in outdoor air than on the AHU filters. In addition to the top three phyla, the phylum Bacteroidetes was found to be much more abundant in outdoor air sample (35%), as compared to the AHU filter samples (0.7–3%). No statistically significant difference was observed for the relative abundance at the phylum level for AHU samples comparing the vacation and active semester periods (*p*>0.05, ANOVA).

**Fig 7:**
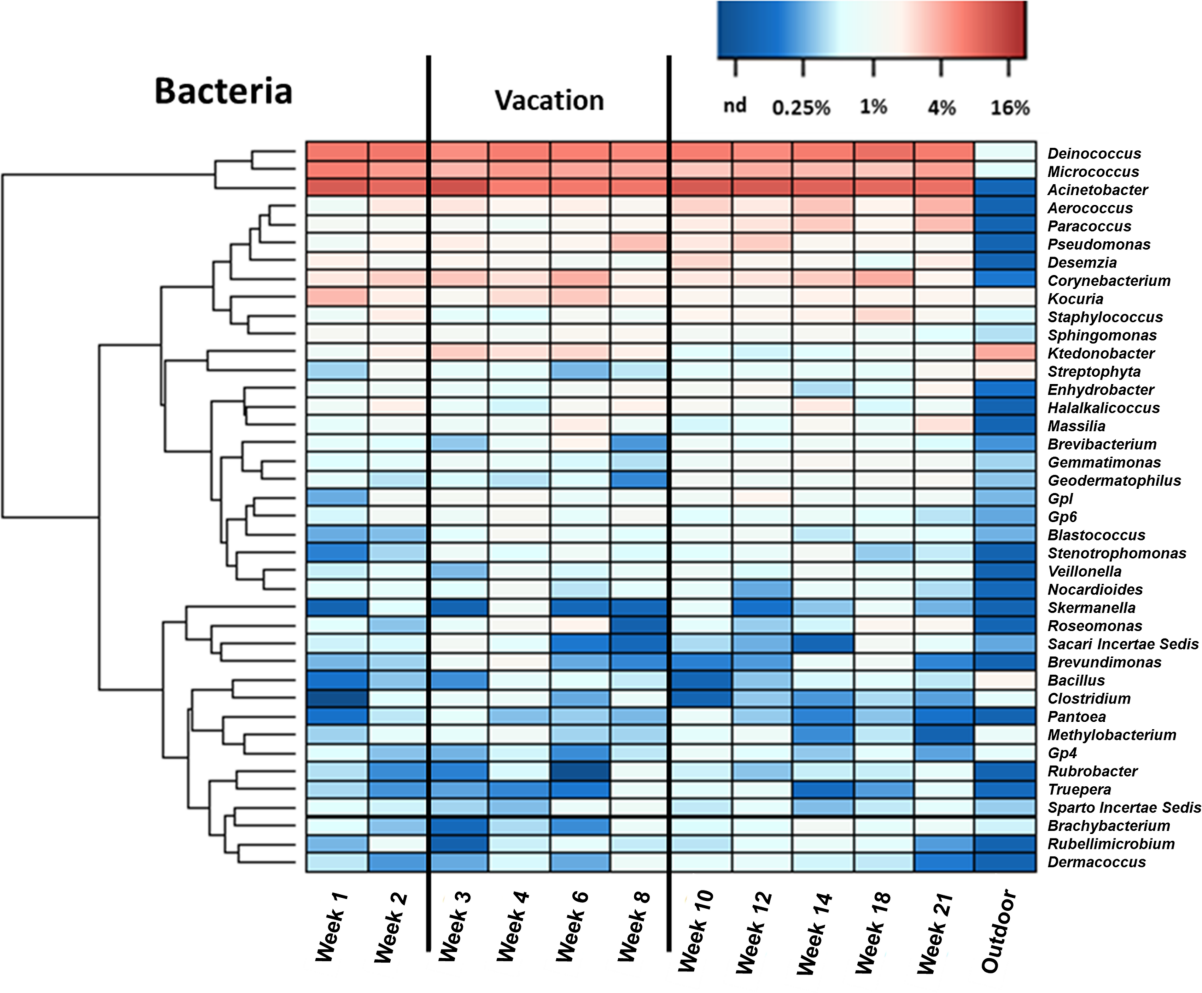
Bacterial genus heatmap. Heatmap composition of the top 40 bacterial genera identified from 11 AHU filter samples and the outdoor reference.

The bacterial genus heatmap (Fig 7) reveals that some of the dominant bacterial genera and their mean relative abundances for the AHU filter samples were *Acinetobacter* (14%), *Deinococcus* (9.4%), *Micrococcus* (5.9%), *Corynebacterium* (3.5%), and *Pseudomonas* (2.2%). In outdoor air, these genera exhibited much lower abundances: 0.1% for *Acinetobacter*, 0.7% for *Deinococcus*, 0.5% for *Micrococcus*, 0.1% for *Corynebacterium*, and below detection for *Pseudomonas*. On the contrary, bacterial genera that were abundant in the outdoor sample, such as *Ktedonobacter* (5.4%), *Streyptophyta* (2.2%), *Methylobacterium* (0.9%) and *Bacillus* (2.0%), comprised only a small percentage of the ventilation-system filter bacteria: 1.6% for *Ktedonobacter*, 0.6% for *Streyptophyta*, 0.5% for *Methylobacterium*, and 0.3% for *Bacillus*. *Kocuria* and *Staphylococcus* were the only genera present at ≥ 1% average abundance in both AHU filter and outdoor samples. *Kocuria* accounted for 2.7% of the AHU filter bacteria and 1.6% of outdoor air bacteria, while the average abundances of *Staphylococcus* were 1.4% on the AHU filter and 1.0% for the outdoor sample. While most bacterial genera were found in both the AHU filter and in outdoor air, the much lower abundance of prominent outdoor genera on the AHU filter suggests that those bacteria detected on the AHU filter primarily originated from indoor sources and were carried by the recirculated indoor air to the filter. This finding is consistent with quantitative analysis results and with expectations based on previous studies that highlight human occupants as sources of indoor airborne bacteria [11,12,26]. Other related work has documented how interior architectural design could affect indoor bacterial communities [30] and how airborne microbes from similar AHU filter analysis, which were found to be primarily bacteria, came mainly from indoor niches instead of from the surrounding outdoor environment [2].

The relative abundance of most bacteria showed no substantial change between the active semester and vacation period. There was no significant difference between the bacterial communities of the two periods (both ThetaYC and Jclass metrics, *p*>0.05). Included in this finding are the human-associated bacterial genera (from skin, hair, oral or gut microflora): *Corynebacterium*, *Staphylococcus*, *Veillonella*, *Streptococcus*, *Enterobacter*, *Propionibacteria* and *Fusobacterium* [26], whose relative abundances remained relatively constant throughout the entire study period. The human-related bacterial genera accounted for an average abundance of 6% of total bacteria during the 21-week period and there was only a modest increase (ANOVA *p*>0.05, insignificant) in average abundance during the active semester as compared with the vacation period. This finding, combined with the evidence presented in Fig 3, suggests that the intensity of human occupancy and associated activity may affect the indoor airborne bacterial quantities but not so much the bacterial composition in indoor air. *Ktedonobacter* was the only genus showing significant change in abundance (*p*<0.05 ANOVA) between the vacation (2.8% of total bacteria) as compared to the active semester period (0.9% of total bacteria). As *Ktedonobacter* was the most abundant genus found in the outdoor reference, we suspect that the relative strength of the outdoor influence increased as occupancy level decreased during the vacation time.

Toward evaluating the use of AHU filters as an indoor bioaerosol sampler, the microbial diversity analyses reported in this study show how the recirculated indoor air could influence the microbial composition of biomass found on the filter. With an outdoor reference, an indoor air study based on AHU filter sampling would be able to distinguish which bacteria, fungi or other biological materials are predominantly brought to the filter from the served indoor environment.

As an additional caution, the relatively consistent community structure (ThetaYC, *p*>0.05) of both bacterial and fungal assemblages during the relatively long 21-week monitoring period highlights the robustness of the sampling approach for microbial profiling of the served indoor air. There is, however, a potential to overestimate or underestimate certain microbes which, due to their specific physical or chemical properties, are either more or less prone to withstand the challenges associated with AHU filter sampling, such as shear-induced stresses, variable temperature and humidity, and long sampling periods. Organisms that are resilient to such conditions could tend to become more abundant, due to consistent contribution from the air or possible growth on the filter medium, leading to underestimation of other organisms. For this reason, we recommend that future studies involving AHU filter sampling also investigate how durable the target organisms are relative to sampling-related stresses.

## Conclusion

This is the first study to present empirical evidence about how DNA accumulates with time on a normally functioning AHU filter in a modern building situated in a tropical climate. Under conditions relevant to a university library, with substantial occupancy and a high proportion of recirculated air passing through the air handling system, bacterial DNA appears to originate primarily from the indoor environment in association with occupancy. The bacterial DNA exhibited low similarity to the composition of outdoor air and accumulated at rates that correlated strongly with occupancy level. No significant difference in bacterial community composition was found as occupancy changed.

In contrast, it appears that the outdoor influence was stronger for fungi on the ventilation system filters. Both the AHU filter and the outdoor reference sample shared similar composition with regard to the most abundant fungal genera. The accumulation rate of fungal DNA did not vary with indoor occupancy level. There was, however, a small group of previously reported human-related fungal genera (up to 7% average abundance), which exhibited a significant difference in relative abundance between the low and high occupancy conditions.

Total DNA accumulation showed behaviour intermediate to those of the bacteria and fungi. In particular, the accumulation rate showed some correlation, albeit not statistically significant, with human occupancy.

Our study also pointed out the possibility that DNA is not consistently conserved on AHU filters during the months-long period of typical use. A substantial dip in the accumulation of fungal DNA was observed during a multiweek period in the middle of the sampling campaign. This decline was associated with a reduction in the outdoor air intake of the ventilation system.

Despite this decline, our results indicated that the microbial community structure was generally maintained. With a complementary outdoor reference sample, such a finding highlights the applicability of AHU filter sampling in probing the microbial assemblages of the served indoor air. However, in terms of quantitative analysis, the case of DNA loss from AHU filters warrants future investigation to understand the causes and the extent of such loss. If DNA degrades while on a filter, then there is a need to understand the role of such degradation in quantitative bioaerosol studies. For example, quantitative comparisons among filter samples from different AHUs with similar operating parameters would be acceptable but comparing AHU filter samples to other air samples collected with relatively milder sampling conditions (e.g. lower flowrate or shorter duration) must be performed cautiously as the AHU filter samples may underestimate actual airborne concentrations differently than the other types of samplers.

Moving forward, to gain deeper understanding in bioaerosol dynamics in ventilation systems in general and on AHU filters in particular, we recommend that future studies focus on a comprehensive comparison between direct indoor air sampling in comparison to AHU filter sampling. We also suggest a need for laboratory-based studies that would investigate more systematically and under better control the topic of DNA conservation on AHU filters.

## Supporting information

S1 File Sampling activity schematic diagram, α and β diversity analysis. (XLSX)

S2 File Report on PCR inhibition check for AHU filter samples. (DOCX)

S3 File DNA conservation experiments for AHU filters. (DOCX)
