# Supplementary material for: Exploring temporal patterns of bacterial and fungal DNA accumulation on a ventilation system filter for a Singapore university library
Source: PLoS One. 2018 Jul 18;13(7):e0200820. doi: 10.1371/journal.pone.0200820 (PMC6051664; doi:10.1371/journal.pone.0200820)
Supplement: S2 File — (DOCX) [file pone.0200820.s002.DOCX]

Supplementary document for manuscript:

**Time Series of DNA Accumulation on a Ventilation System Filter in a Singapore University Library**

**S2 File: Report on PCR Inhibition Check for AHU Filter Samples**

Irvan Luhung*^1,2^, Yan Wu^1,5^, Siyu Xu^4^, Naomichi Yamamoto^4^, Victor Wei-Chung Chang^1,6^, William W Nazaroff^1,3^

^1^ SinBerBEST Program, Berkeley Education Alliance for Research in Singapore (BEARS), Singapore

^2^ Singapore Centre for Environmental Life Sciences Engineering (SCELSE), Nanyang Technological University, Singapore

^3^ Department of Civil and Environmental Engineering, University of California, Berkeley, CA, USA

^4^ Department of Environmental Health Sciences, Graduate School of Public Health, Seoul National University, Seoul, South Korea

^5^ School of Environmental Science and Engineering, Shandong University, Jinan, China

^6^ Department of Civil Engineering, Monash University, Victoria, Clayton, Australia

** Corresponding email:* [irva0003@ntu.edu.sg](mailto:irva0003@ntu.edu.sg)

**Introduction**

The presence of polymerase chain reaction (PCR) inhibitors in a quantitative PCR (qPCR) assay could cause an underestimate, potentially serious, of quantified DNA concentrations. Inhibitors are common in environmental samples and might occur in the air handling unit (AHU) filter samples in this study, either because of the properties of the filters themselves or because the filters have collected airborne dust from various sources. Indeed, the dip in the abundance of fungal DNA exhibited between weeks 6 and 10 (Fig S1) could conceivably be influenced by the existence of certain PCR inhibitors that were not properly removed during the DNA extraction step.


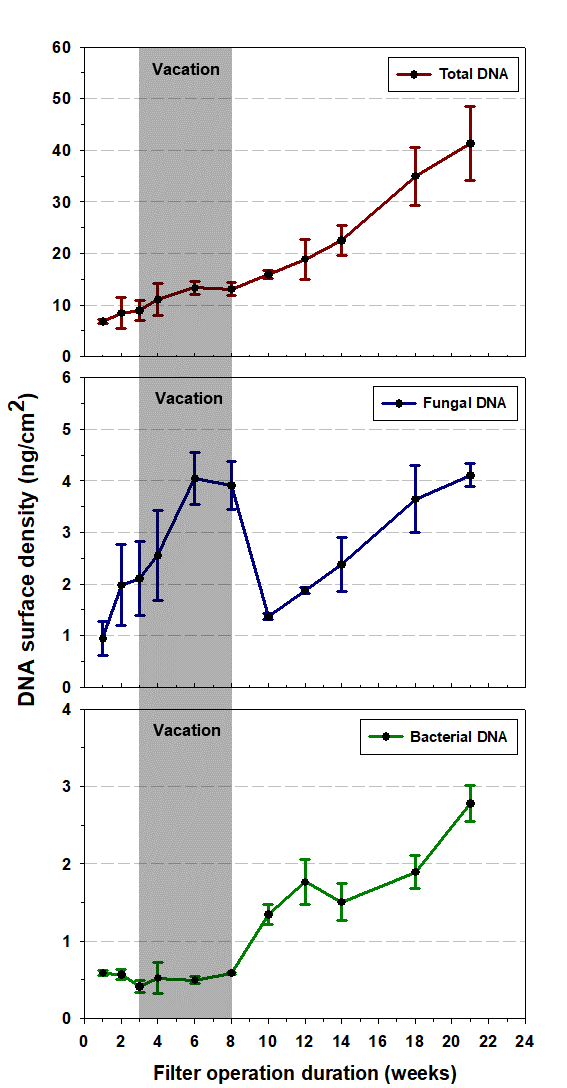


**Fig S1. Time-series profile of DNA accumulation on AHU filter.** Total (red), fungal (blue) and bacterial (green) DNA surface densities sampled over the course of the 21-week study period. The error bars represent standard deviations based on three biological replicates.

In this supplement, we report on the results of PCR inhibition check experiments as applied for the assessment of AHU filter samples, particularly focusing on the weeks involved in the unexpected fungal DNA dip. The chosen samples for more detailed assessment are week 6, week 10 and week 12. The finding from this effort is that the observed trend, in which fungal DNA decreased substantially from week 6 to week 10 and then increased from week 10 to week 12, is not believed to have been caused by inhibition.

**Method**

PCR inhibition was investigated by performing dilutions on the DNA samples. As illustrated in Fig S2, in the case where there is no inhibition, if DNA concentration is diluted (reduced) and qPCR is performed, then the amplification curve should become evident cycles later, i.e., increasing the cycle threshold (*Ct*) value. For example, with 100% qPCR efficiency, if one dilutes the DNA concentration by a factor of 10, then the *Ct* value is expected to increase by 3.3 cycles (since 10 = 2^3.3^). Calculating based on the standards, our qPCR efficiencies were 84% for fungi and 86% for bacteria. Accounting for these efficiency values and possible pipetting accuracy limitations, we expected a change of *C*t value in the range of 3.4 to 3.7 cycles for 10× dilution.


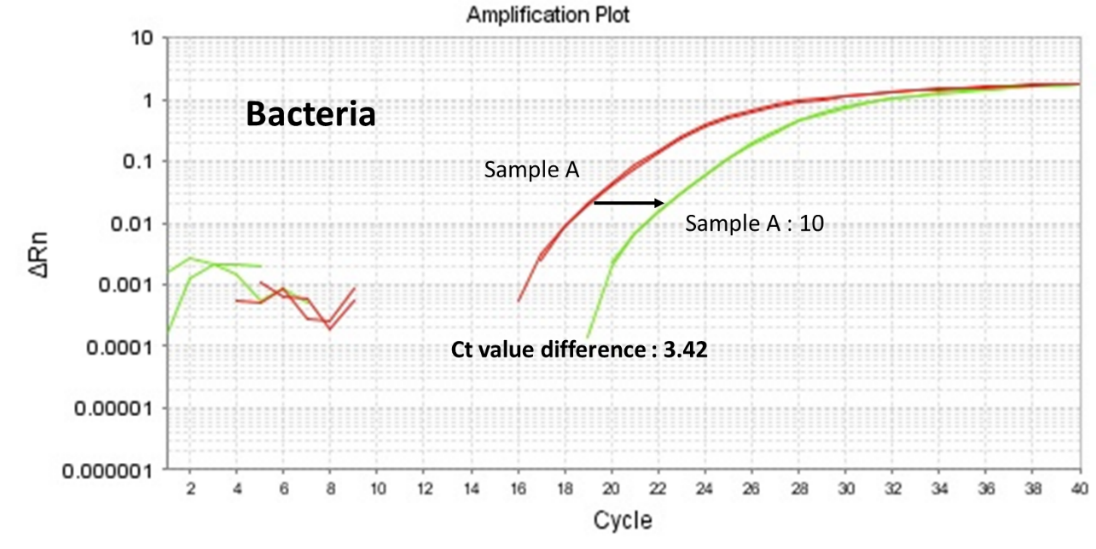


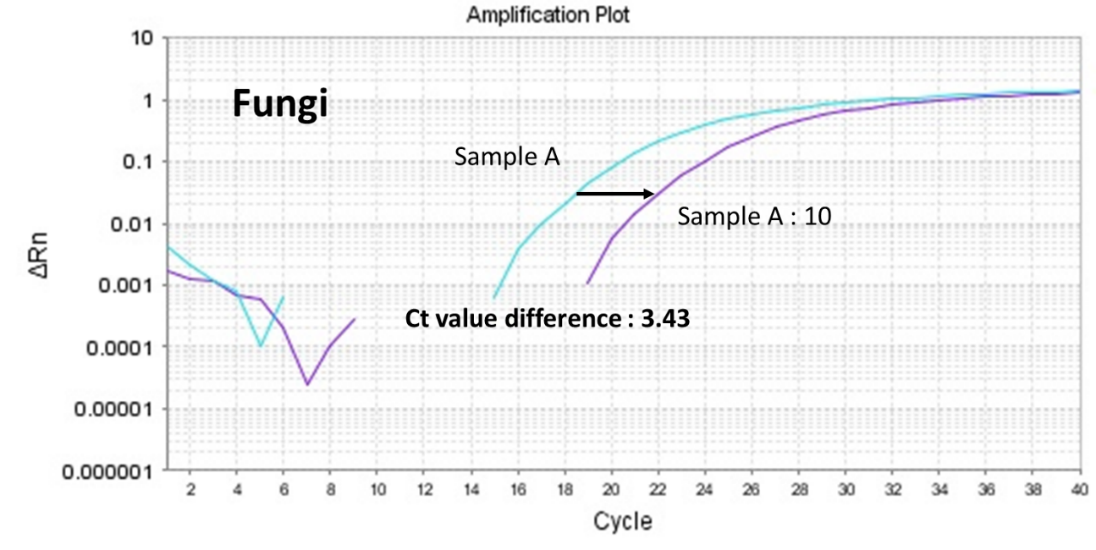


**Fig S2. qPCR amplification curves for uninhibited DNA sample.** Bacterial DNA qPCR (top) for sample A (red line) and its diluted sample (green line). Fungal DNA qPCR (bottom) for sample A (blue line) and its diluted sample (purple line).

In a case in which there is significant inhibition (Fig S3), when the DNA concentration is diluted and qPCR is performed, the amplification curve will transition earlier. The reason is that the effect of diluting the inhibitor is more favorable to the PCR reaction than the reduction of the DNA concentration itself.


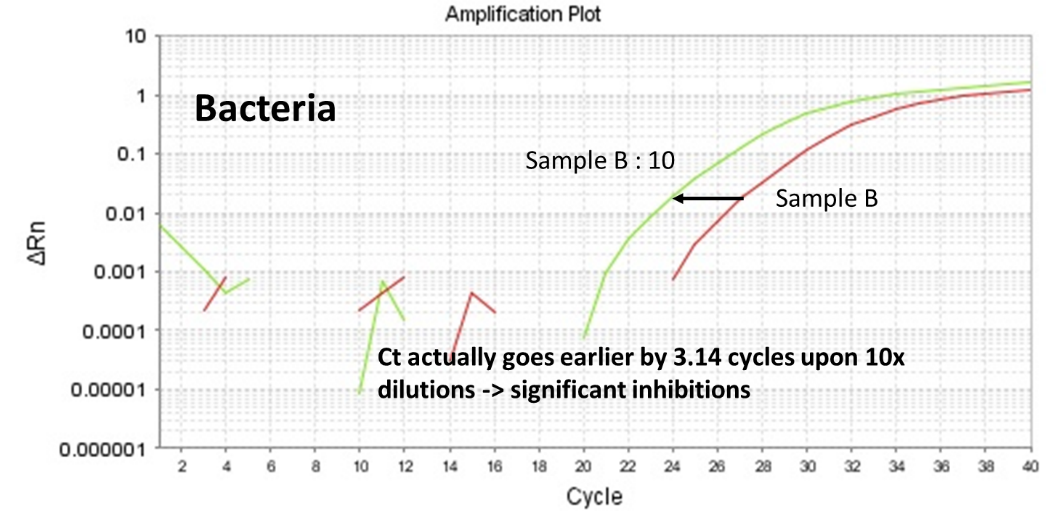


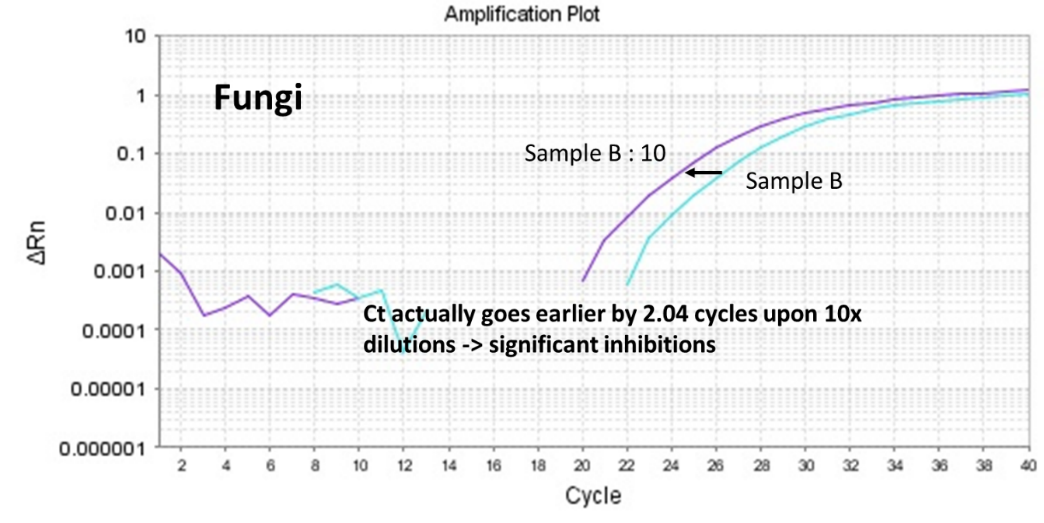


**Fig S3. qPCR amplification curves for inhibited DNA sample.** Bacterial DNA qPCR (top) for sample B (red line) and its diluted sample (green line). Fungal DNA qPCR (bottom) for sample B (blue line) and its diluted sample (purple line).

In a case such as shown in Fig S3, although there are ways to purify the DNA sample so as to remove the inhibitor, we decided to exclude DNA from further analysis and perform re-extraction. All the data shown in the main manuscript were found to be free of inhibitor effects based on the test method described in this section. In the subsequent results section of this supplement, to document that the sudden dip in fungal DNA is not because of inhibition, the test results for samples from weeks 6, 10 and 12 are shown.

**Results**

Fig S4 displays the qPCR amplification curves for bacteria for weeks 6, 10, and 12, along with the results for 10× diluted samples. The results conform to the trend shown in Fig S1 for bacterial DNA, in which the DNA concentration increased (corresponding to a decreasing *Ct* value) from week 6 to week 12. The changes in *Ct* values for the 10× diluted samples were consistently within the 3.4-3.7 range, indicating that there is no significant inhibition in the three samples.


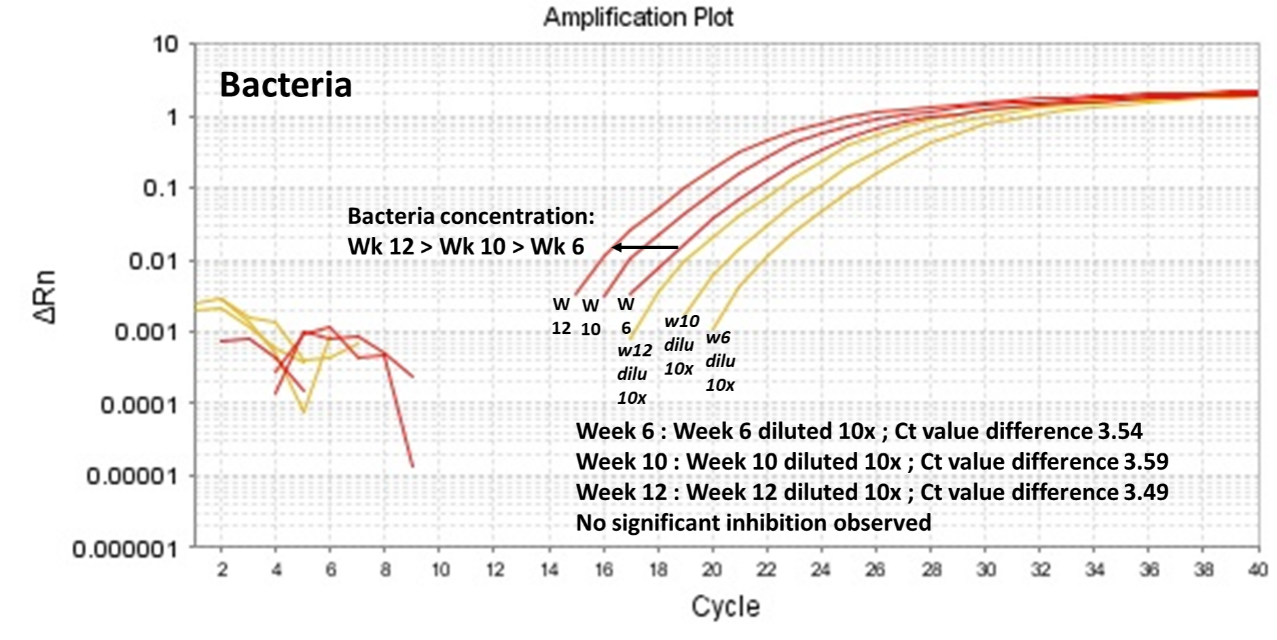


**Fig S4. Bacterial DNA qPCR amplification curves for samples from weeks 6, 10 and 12.** qPCR amplification curves for samples from weeks 6, 10 and 12 (orange-red lines) complemented by the amplification curves of the corresponding diluted samples (yellow-orange lines).

Fig S5 displays the fungal DNA qPCR results from the same samples. Consistent with the trend shown in Fig S1, fungal DNA exhibits a reduction in abundance from week 6 to 10 (as evidenced by an increasing *Ct* value), followed by an increase in DNA concentration (a corresponding decrease in *Ct* value) from week 10 to 12. The respective increases in *Ct* values for each diluted sample was in the range of 3.4-3.6, indicating that there was no significant inhibition effect in these qPCR reactions.


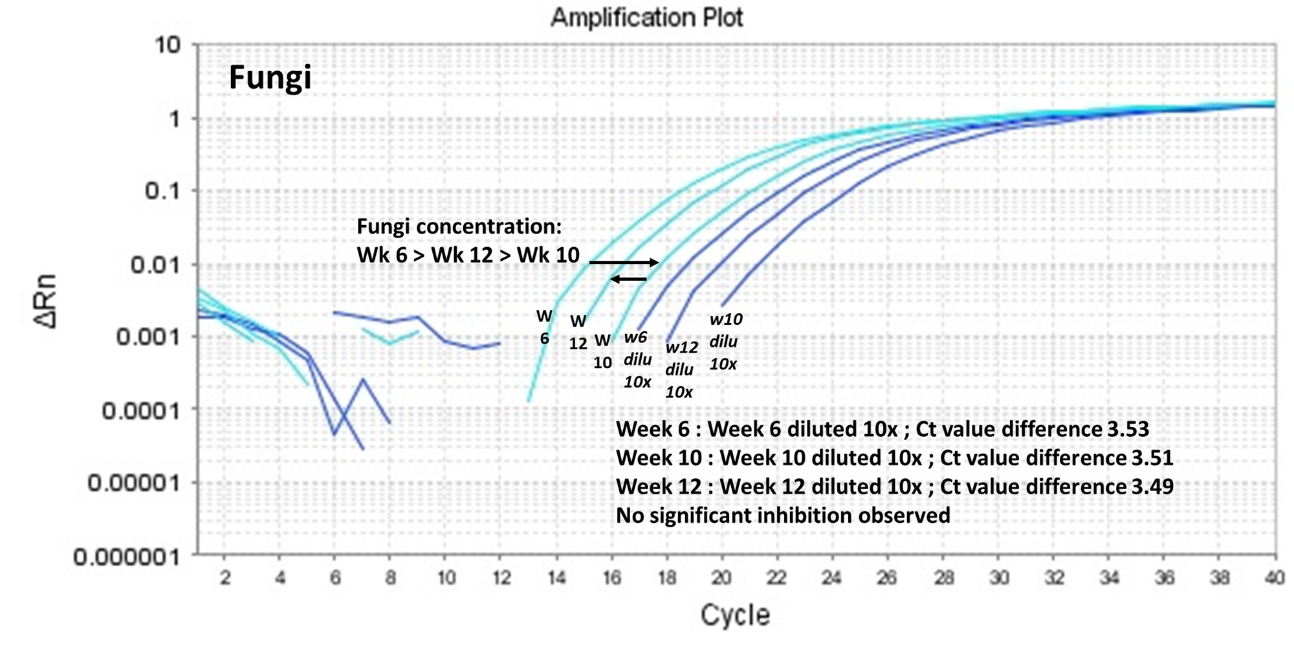


**Fig S5. Fungal DNA qPCR amplification curves for samples from weeks 6, 10 and 12.** qPCR amplification curves for samples from weeks 6, 10 and 12 (light blue lines) complemented by the amplification curves of the corresponding diluted samples (dark blue lines).

In addition to the inhibition check results, three additional reasons support the conclusion that the dip in fungal DNA was not caused by inhibition. The first reason is because the dip only happened in fungal DNA. If there was significant inhibition in the sample, a substantial dip in bacterial DNA would also likely be observed as the inhibitor would have affected all PCR reactions, instead of just that for fungi. Second, the same DNA stocks were submitted for amplicon sequencing which also involves PCR reactions. All samples were successfully sequenced, a fact that argues against inhibition. Third, after the reduction (exhibited in both week 8 and week 10 samples) the subsequent increase in fungal DNA occurred at a relatively steady pace following the concentration from week 10 to week 12 onwards. The consistency of the pattern other than the period of the dip supports an inference that inhibition did not materially affect the results.

**Conclusion**

Based on these results, we conclude that the dip in fungal DNA is not caused by inhibition in the PCR reaction.
