# Supplementary material for: Exploring temporal patterns of bacterial and fungal DNA accumulation on a ventilation system filter for a Singapore university library
Source: PLoS One. 2018 Jul 18;13(7):e0200820. doi: 10.1371/journal.pone.0200820 (PMC6051664; doi:10.1371/journal.pone.0200820)
Supplement: S3 File — (DOCX) [file pone.0200820.s003.DOCX]

Supplementary document for manuscript:

**Time Series of DNA Accumulation on a Ventilation System Filter in a Singapore University Library**

**S3 File: DNA Conservation Experiments for AHU Filters**

Irvan Luhung*^1,2^, Yan Wu^1,5^, Siyu Xu^4^, Naomichi Yamamoto^4^, Victor Wei-Chung Chang^1,6^, William W Nazaroff^1,3^

^1^ SinBerBEST Program, Berkeley Education Alliance for Research in Singapore (BEARS), Singapore

^2^ Singapore Centre for Environmental Life Sciences Engineering (SCELSE), Nanyang Technological University, Singapore

^3^ Department of Civil and Environmental Engineering, University of California, Berkeley, CA, USA

^4^ Department of Environmental Health Sciences, Graduate School of Public Health, Seoul National University, Seoul, South Korea

^5^ School of Environmental Science and Engineering, Shandong University, Jinan, China

^6^ Department of Civil Engineering, Monash University, Victoria, Clayton, Australia

** Corresponding email:* [irva0003@ntu.edu.sg](mailto:irva0003@ntu.edu.sg)

**Introduction**

We explore whether the dip in fungal DNA concentration from week 6 to week 10 (see Fig S1) could have had a contribution from DNA not being conserved on the air handling unit (AHU) filter. During the relatively long period of air treatment by the filters (up to 3 months in this study), it is feasible that losses occur, e.g. owing to the dislodging of previously collected bioaerosol material from the filter, or because of DNA degradation caused by environmental stresses, such as periods of elevated temperature or shear induced by air flow.


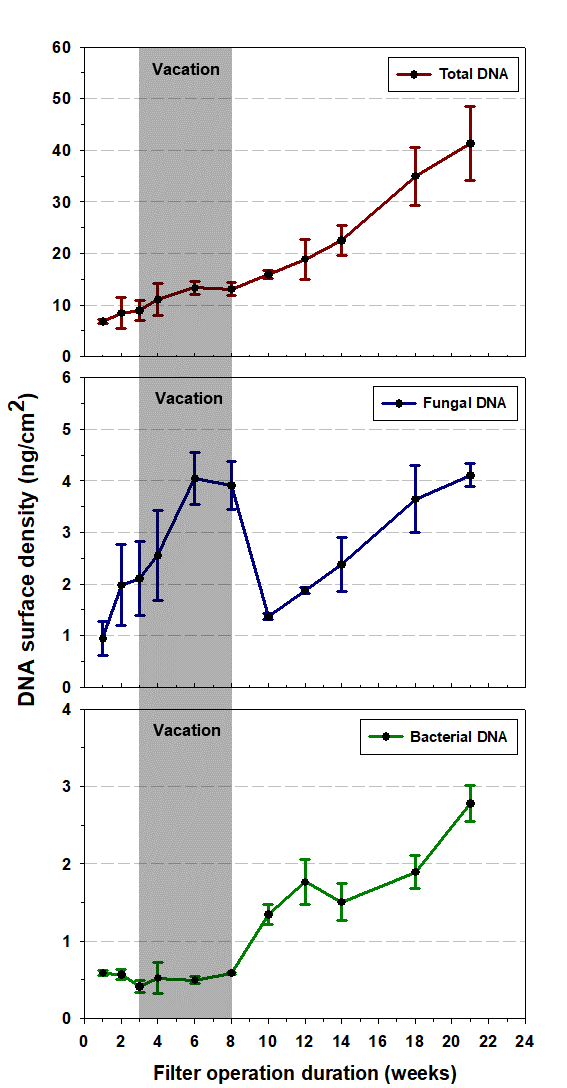


**Fig S1. Time-series profile of DNA accumulation on AHU filter.** Total (red), fungal (blue) and bacterial (green) DNA surface densities sampled over the course of the 21-week study period. The error bars represent standard deviations based on three biological replicates.

In this supplement, we report the results from supporting experiments that were conducted to test whether DNA is conserved on AHU filter samples. In these tests, samples of the used AHU filter were exposed to elevated temperature in a controlled chamber or to a flow of clean air. DNA concentrations were measured before and after the treatments to judge whether a portion of DNA could have been lost owing to the specific treatment conditions.

**Materials and Methods**

Two sets of used AHU filter pieces were collected each month from the same AHU as studied in the main project. In all, there were three collection sessions spanning a typical 3-month period of AHU filter deployment. After collection, the filter sets were exposed to one of two conditions. The first set was put into a temperature-controlled chamber maintained at 35 °C and 45% RH for one week. The other set was put into a customized air flow test rig. The test rig consists of an in-line fan, an upstream HEPA filter and the test filter. In this rig, the air would first pass through the HEPA filter before reaching the test filter with the fan positioned further downstream. This configuration allows only clean air to pass through the test filter and is not expected to add particulate matter from the surrounding environment to the test filter during the week-long exposure interval. This test allows us to discern whether air flow, at a fixed speed of 1.5 m/s, adversely influences the already captured biomass on the test filter. The temperature and air speed for these two test conditions were chosen based on actual temperature and air flow speed measurements in the AHU room from which the filters were acquired. Based on sensor readings inside the AHU, the relative humidity was relatively constant within the range of 61-65% both when the AHU was on and off. The temperature, on the other hand, fluctuated according to the operation of the AHU. When the AHU was on, the temperature was well kept in a relatively small range of 23.5-24 °C. When the AHU was turned off, the temperature gradually increased over night, reaching a peak in the range 31-35 °C, depending both on the outdoor temperature and on whether or not the dehumidifier was recharging its dessicant. The temperature then quickly dropped back to 24 °C (within 5-10 minutes) as soon as the AHU resumed operation in the morning. Due to a large portion of recirculated indoor air being drawn into the AHU, CO2 concentration followed the trend of occupancy in the indoor environment, ranging from ~600 ppm during low occupancy to ~1100 ppm during high occupancy.

DNA was extracted from the filter pieces before and after treatment with the same method as described in the main manuscript. The extracted DNA was analysed with fluorometry (Qubit) for total DNA concentration and with qPCR for bacterial and fungal DNA concentrations. The paired t-test was performed to determine whether the difference between the before and after treatment samples are statistically significant at *p* < 0.05.

**Results and Discussion**

The results, displayed in Fig S2, indicate a reduction in DNA concentration of all three measured biological entities on the filter after the week-long temperature treatment at 35 °C and after week-long exposure to a 1.5 m/s clean air flow. On average, the temperature treatment induced 40% reduction for total DNA, 26% reduction for fungal DNA and 18% reduction for bacterial DNA, while the air flow treatment induced 40%, 24% and 12% reduction to total, fungal and bacterial DNA respectively.

For total and fungal DNA, the reduction of DNA appeared to be more prominent on the month 1 and month 2 filter samples with an average of 47% reduction for total DNA and 29% reduction for fungal DNA (all *p* < 0.05). The month 3 filter samples, on the other hand, showed more modest DNA reductions, averaging 28% for total DNA, with only the airflow treatment showing a statistically significant difference, and 17% reduction for fungal DNA, with both treatments showing statistically insignificant difference (both *p* > 0.05). This evidence is consistent with a plausible explanation that, as the filter keeps processing air, the proportion of biomass that is able to persist in response to the environmental challenges would tend to become bigger. Conversely, the portion of biomass that could not withstand these conditions becomes smaller with time. Compared to fungal DNA, the bacterial DNA indicated a more modest and stable reduction of DNA across the three filter samples and the two treatments, with reductions in the range of 11-20%.


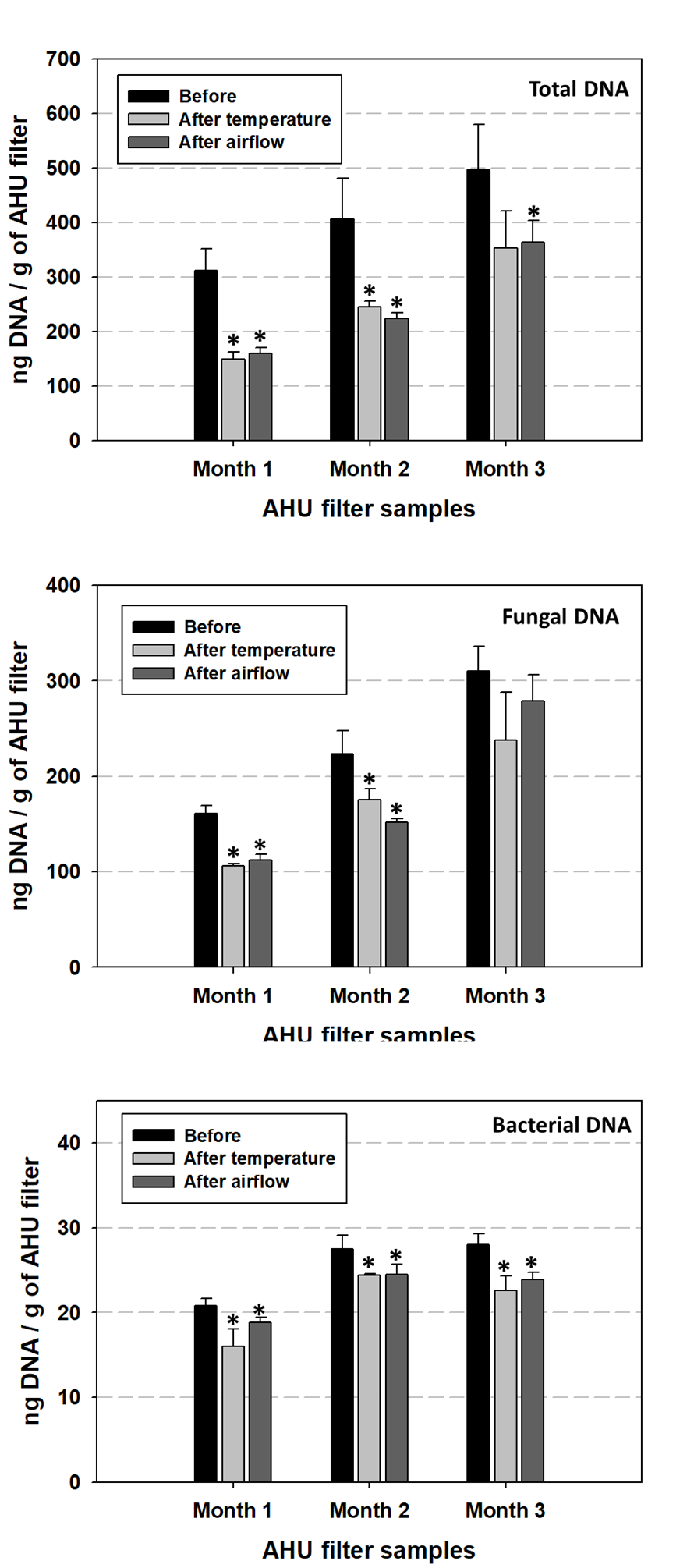


**Fig S6.** DNA measurements for total DNA (top), fungal (middle) and bacterial DNA (bottom) before the treatment (black bar), after the 35 °C incubation (light grey bar) and after the 1.5 m/s airflow treatment (dark grey bar) for one week each. The error bar represents the standard deviation from the three replicates and * denotes a statistically significant difference between the before-treatment and the after-treatment samples (*p* < 0.05).

The AHU in the library operates 12 hours per day. As it samples the air, it is possible that the filter gains some biomass from the air and simultaneously loses some previously collected biomass owing to shear caused by this air flow or to associated vibrational motion. When the AHU is off, the temperature in the AHU room rises to a peak of ~ 35 °C because of heat sources, including intrusion of warm outdoor air and the internal use of a heater designed to recharge a desiccant dehumidifier. Based on these preliminary experimental results, continuous exposure to such temperature and air flow over the course of the filter operation (up to 3 months) could serve to attenuate the DNA signal associated with biomass accumulated on the filter.

**Conclusion**

In the context of studying indoor bioaerosols, the results indicate that part of the DNA may be lost during the deployment period of the AHU filter. It is important to assess how durable target organisms are to the selected sampling approach, especially if quantitative analysis is intended. It would be important for the future to better understand the mechanism of DNA loss from AHU filters as it could also have impacts on building occupants in the corresponding indoor environment or for downstream equipment designed to be protected by the filter.
